# Deficit irrigation improves phenolic content and antioxidant activity in leafy lettuce varieties

**DOI:** 10.1002/fsn3.559

**Published:** 2017-12-06

**Authors:** Dunsfort N. Malejane, Peter Tinyani, Puffy Soundy, Yasmina Sultanbawa, Dharini Sivakumar

**Affiliations:** ^1^ Phytochemical Food Network Research Group Department of Crop Sciences Tshwane University of Technology Pretoria South Africa; ^2^ Centre for Food Science and nutrition Queensland Alliance for Agriculture and Food Innovation (QAAFI) The University of Queensland Coopers Plains QLD 4108 Australia

**Keywords:** Abiotic stress, ascorbic acid, chlorogenic acid, fresh mass, kaempferol, *Lactuca sativa* L.

## Abstract

This study investigated the effect of deficit irrigation at three management allowable depletion levels (MAD) 25%, 50%, and 75% on phytochemicals, ascorbic acid, antioxidant activity, glucose, fructose, and fresh and dry mass in two green leafy lettuce cultivars (Lollo Bionda and Vera) at harvest. Genotype response was observed during deficit irrigation on fresh and dry mass at harvest. Vera revealed similar fresh mass at MAD levels 25% and 50%. Chicoric acid, caftaric acid, and chlorogenic and caffeic acid tended to increase at 50% MAD, while kaempferol, quercetin, and myricetin increased at 75% MAD in both cultivars indicating increasing antioxidant properties. Ascorbic acid content declined with increasing MAD levels and no definite trend on β‐carotene content was noted in these cultivars with respect to MAD irrigation. Deficit irrigation at 50% and 70% increased glucose concentration in cv. Lollo Bionda. Deficit irrigation influences the abiotic stress condition that stimulates the biosynthesis of phytochemicals in plants and improves crop quality. However, deficit irrigation can affect marketable crop yield. Based on findings of this study, the lettuce cv. Vera can be recommended as a suitable cultivar for deficit irrigation (at MAD 50% levels) for improving dietary phytochemicals and crop quality without compromising fresh mass for marketing.

## 1 INTRODUCTION

Lettuce (*Lactuca sativa* L.) is becoming an increasingly important vegetable crop in salads. Lettuce is also of particular interest due to its high content in antioxidants and phytochemicals including caffeic acid and its derivatives, flavonols, vitamins C and E, chlorophyll, and carotenoids (Llorach, Martínez‐sánchez, Tomás‐Barberán, Gill, & Ferreres, 2008). Lettuce is one of the vegetables that when highly consumed has proved to have a good contribution to human health Dias and Ryder (2011). There are epidemiological studies that showed that constant consumption of fruits and vegetables as a lifestyle has positive effects on improving human health and prevention of chronic diseases (Tohill, 2004). Deficit irrigation as a water‐saving strategy practice is mostly adopted in arid areas (Yang, Luo, Sun, & Wu, 2015). Lettuce plant is very sensitive to water shortage in the soil (Şenyiğit & Kaplan, 2013) and deficit irrigation (reduced) at 80% and 60% of evapotranspiration was reported to affect leaf number, leaf area index, and dry matter accumulation in lettuce cultivar Royal (Karam, Mounzer, Sarkis, & Lahoud, 2002). Deficit irrigation was shown to influence phenolic compounds and antioxidant properties in different crops (Niculcea et al., 2015; Peña et al., 2013). Intake of dietary phytochemicals such as phenolic and flavonoid compounds have many health benefits due to their antioxidant properties (Liu, 2013). Different variety‐specific responses were reported with respect to anthocyanin, flavonols, and hydroxycinnamic acids in grape varieties “Graciano” and “Tempranillo” subjected to deficit irrigation (Niculcea et al., 2015). Deficit irrigation on water usage parameters such as evapotranspiration, yield response factor, and water use efficiency were studied in detail (Karam et al., 2002). However, information on genotype responses in leafy lettuce cultivars on accumulation of polyphenolic compounds are limited, and implementing appropriate deficit irrigation regimes can improve the dietary phytochemicals and yield at harvest.

Therefore, the objective of this investigation was to evaluate the influence of deficit irrigation at two management allowable depletion levels‐MAD [(25% of control, 50% of control and 75% (the control)] in two green leafy lettuce on i) fresh leaf mass, ii). ascorbic acid and β‐carotene content, ii) hydroxycinnamic acid, chicoric acid, chlorogenic acid, caffeic acid and caftaric acid (2‐caffeoyl‐L‐tartaric acid) and iii). Flavonol (kaempferol, myricetin and quercetin), iv). Antioxidant property, v) glucose and fructose at harvest.

## 2 MATERIALS AND METHODS

### 2.1 Description of trial site

Lettuce (cv. Lollo Bionda and Vera) seeds were obtained from Starke Ayres Seed (Pty) Ltd. Field trials were carried out at the experimental farm (25°35′ S; 28°21′ E; 1,164 m a.s.l), Pretoria, South Africa. Seeds were planted in seed trays and the developing seedlings were irrigated twice a day till they had developed two full leaves and fertigated once a day with Multifeed (5 g in 5 L of water), a foliar fertilizer. Seedlings were planted in soil at fully developed two‐leaf stage. During transplanting based on Soundy et al. (2005), preplant fertilizer (13N, 0P, 10.8K) was added at the rate of 230 kg/ha, and after transplanting, 150 mg/L from 20N, 8.6P, and 16.7K as a start fertilizer. Also, after a week of transplanting, 150 kg N/ha and 180 kg K/ha were applied. Copper count N (Hygrotech (Pty) Ltd.) at a concentration of 2 ml in 16 L of water was adopted as foliar application to control pests. Spore kill (8 ml in 16 L water) was used to control diseases (Ntsoane, Soundy, Jifon, & Sivakumar, 2016).

A 2 × 2 × 2 factorial experiment was laid out in a randomized complete block design, with three replications. The following factors were considered: two lettuce cultivars (Lollo Bionda and Vera) and three irrigation regimes applied as MAD of available soil water content (25% treatment control; 50% and 75% MAD). Thus, the experiment consisted of 6 treatments and 18 experimental units. The irrigation regimes were set and monitored with the aid of neutron probe measurements, which were taken at least three times per week. Depleted soil water content was typically replenished three times, twice and once a week for the 25%, 50%, and 75% depletion levels of available soil water content, respectively. This was conducted through drip irrigation, in which water was applied at a rate of 2 L/hr.

### 2.2 Experimental design and treatments

#### 2.2.1 Fresh mass

Harvesting commenced at 6 weeks after transplanting. During each harvest, fresh mass was determined by picking fresh marketable leaves. After each harvest, the remaining leaves were allowed to regrow for the next harvest. In order to obtain accurate results, the plants were weighed in the field to avoid loss of water.

#### 2.2.2 Chemicals and reagents

Authentic standards for chlorogenic acid (purity ≥ 98.0%), caffeic acid (purity ≥ 98.0%), caftaric acid (purity ≥ 97), chicoric acid (purity > 98.0%), quercetin (purity ≥ 95.0%), kaempferol (purity ≥ 90.0%), epicatechin (purity ≥ 98.0%), myricetin (purity ≥ 99.0%), β‐carotene (≥95%), sucrose (≥99%), glucose (≥99.5%) HPLC‐grade solvents (0.1% [v/v] formic acid and water, methanol [≥99%], ethanol ≥99.8% methyl *tert*‐butyl ether [MTBE]), ethyl acetate, acetone, hexane 2,6‐dichlorophenolindophenol, ascorbic acid, metaphosphoric acid, gallic acid, 2,2‐diphenyl‐1‐picrylhydrazyl, 2,4,6‐tripyridyl‐*s*‐triazine solution, ferric chloride (FeCl_3_.6H_2_O), HCl, ribitol, methoxyamine hydrochloride, pyridine, *N*‐methyl‐*N*‐(trimethylsilyl) trifluoroacetamide were purchased from Sigma Aldrich (Johannesburg, South Africa).

#### 2.2.3 Ascorbic acid content

Ascorbic acid content determination was performed by using 2,6‐dichlorophenolindophenol dye in a titrimetric method using 5 g of fresh lettuce leaves (Ntsoane et al., 2016). The results were expressed as mg/kg on a fresh weight basis.

#### 2.2.4 Phenolic acids and flavonols

Hydroxycinnamic acids, such as *chicoric*,* chlorogenic*,* caffeic*, and *caftaric* acids, were determined according to the method described by Tomás‐Barberán, Gil, Castáner, Artés, and Saltveit (1997). Freeze‐dried samples (5 g) were homogenized in 25 ml ethanol–water mixture (4:1 v/v) and extracted using an ultrasonic extraction device (80 kHz, 45°C) (Ntsoane et al., 2016). The resulting mixture was centrifuged at 3,000*g*, filtered, and afterward the ethanol was evaporated from the filtrate at 50°C under reduced pressure using a rotary evaporator. The pigments and the lipids in the mixture were removed by extracting the concentrated mixture dissolved in water (25 ml) with ethyl acetate, and thereafter, the resulting extract was concentrated using nitrogen flow at 20°C (Tomás‐Barberán et al., 1997). The concentrated extract was dissolved in methanol (1.5 ml) and 35 μl filtered via a Nylon syringe filter (0.45‐μm pore size) and 10 μl injected three times for high‐performance liquid chromatography (HPLC) (with an ultraviolet detector, C18 column [100 × 4.6 mm; 5 μm particle size], Model Flexar^™^ 89173‐556 PerkinElmer, Waltham, MA, USA). The mobile phase conditions were maintained according to Tomás‐Barberán et al. (1997). Identification and quantification of phenolic acids were carried out at a wavelength 265, 280, and 320 nm for phenolic acids by comparing peak retention times with those of authentic standards.

Flavonols were identified and quantified using HPLC according to the method described by Llorach et al. (2008) and Ntsoane et al. (2016) using freeze‐dried leaf samples homogenized in 15 ml of methanol–water–formic acid mixture (25:24:3, v/v/v). The separation was obtained on a Col‐Analytical C18 column (100 × 4.6 mm; 5 μm) and the conditions and the mobile phase, flow rate, and gradient elution program were according to Ntsoane et al. (2016). Chromatogram was read at 320, 368, 356, and 520 nm, and the flavonols were identified and quantified using pure standards. Results are reported in mg/kg on a dry weight basis.

#### 2.2.5 β‐Carotene

Estimation of β‐carotene was performed using freeze‐dried leaf samples (5 g) and β‐carotene was extracted using 1.5 ml acetone–hexane mixture (4:6 volume/volume, v/v), and the procedure was adopted was similar to Ntsoane et al. (2016). The absorbance of the filtrate was measured at A663, A505, and A453. The β‐carotene was determined using the following calculation: β‐carotene = 0.216 A663 − 0.304 A505 + 0.452 A453, and was expressed as mg/kg on a dry weight basis.

#### 2.2.6 Antioxidant property

Ferric reducing antioxidant power (FRAP) assay was carried out according to the method described by Wang, Zheng, Khuong, and Lovatt (2016) without modification using FRAP solution (0.3 mmol/L sodium acetate [pH 3.6], 10 mmol/L 2,4,6‐tri pyridyl‐2‐triazine [TPTZ] and 20 mmol/L FeCl_3_ [10:1:1 v/v/v]). The calibration curve was obtained using the Trolox solution at concentration from 10 to 250 mg/L for quantification of FRAP antioxidant activity and the results were expressed as kmol Trolox equivalents antioxidant capacity (TEAC) per kg dry weight (kmol TEAC/kg).

#### 2.2.7 Glucose and fructose contents

Glucose and fructose were estimated following the method of Roessner, Wagner, Kopka, Trethewey, and Willmitzer (2000) with some modifications as described by Glowacz, Bill, Tinyane, and Sivakumar (2017). Freeze‐dried sample (100 mg) was dissolved in 1.4 ml of 100% methanol and 50 μl of internal standard (2 g/L ribitol [w/v] in water) was added. The mixture was agitated for 10 s and subsequently extracted for 15 min at 70°C. Samples were then centrifuged at 6,000*g* for 10 min, and supernatant was mixed with 750 μl of dichloromethane and 1.5 ml of dH_2_O_2_. Afterward, the mixture was centrifuged again at 6,000*g* for 10 min. Thereafter, 150 μl of the upper (polar) phase was evaporated under nitrogen gas to concentrate the sample. For identification and quantification, the residue was derivatized for 2 hr at 37°C in 40 μl of 20 g/L methoxyamine hydrochloride in pyridine. This was followed by a 30‐min incubation with 70 μl *N*‐methyl‐*N*‐(trimethylsilyl) trifluoroacetamide at 37°C. After derivatization, the samples were transferred into injection vials. Derivatized sugars were analyzed using GC/MS with an Agilent J&W DB‐17 (50%‐164 phenyl)‐methyl‐polysil‐oxane column 30 m × 250 μm × 0.25 μm, with helium as a carrier gas at flow rate of 1 ml/min. The GC conditions and run parameters were set up according to Roessner et al. (2000). Glucose or fructose sugar was identified and quantified by comparison of peak area with that of known standard, expressed as mg/g on dry weight basis.

### 2.3 Statistical analysis

Experiments were repeated twice in 2015 and 2016 winter seasons (June–August). The data recorded between the two growing seasons (2015 and 2016) showed similar trends for all variables. Therefore, the 2 years data were pooled and subjected to two‐way analysis of variance to determine the effect of lettuce variety and MAD (25% of control, 50% and 75% MAD) on all the parameters in two lettuce cultivars. Means of significant effects were separated using Fisher's protected least significant differences (LSD) at a 5% significance level. For the quantification of phenolic acids, flavonoids, ascorbic acid, and glucose content, 12 samples per replicate were analyzed.

## 3 RESULTS AND DISCUSSION

**Figure 1:**
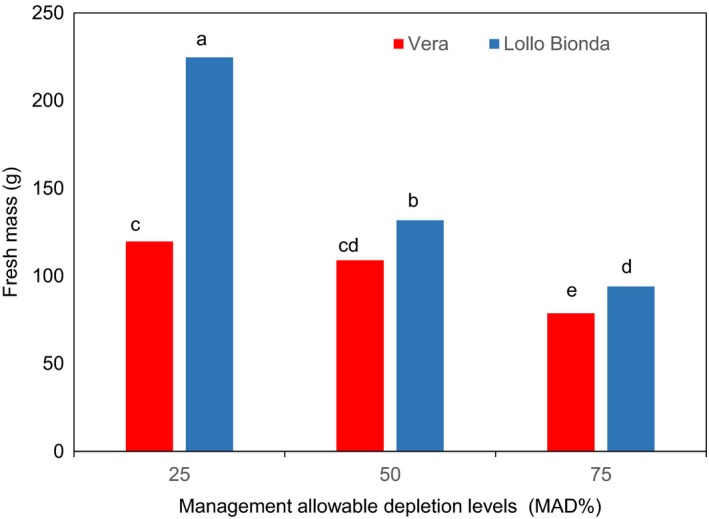
Effect of management allowable depletion levels (MAD%) on fresh mass of green leafy lettuce varieties Vera and Lollo Bionda. Management allowable depletion levels (MAD%): 25% (control), 50%, and 75%. Means in each bar for a specific parameter with the same letter are not significantly different at *p* < .05, Fisher's least significant difference (*n* = 12)

Interaction between the cultivar and the deficit irrigation (MAD levels) on fresh mass at harvest are illustrated in Figure 1. Overall, the increasing deficit irrigation (MAD levels) impacted negatively on fresh mass of both green leafy lettuce cultivars (Figure 1). Deficit irrigation treatments significantly affected the fresh mass at harvest in cv. Lollo Bionda (Figure 1). Fresh mass for cv. Vera was similar at MAD levels 25% and 50% (Figure 1). Similarly, Gallardo et al. (1996) reported that when romaine (i.e., cos) lettuce was watered with a volume 13% below that required in the field capacity treatment (0.87 × FC) and the control crop watered up to field capacity (FC), there was no remarkable difference in fresh mass at harvest. The reduction in yield (fresh mass) was almost 39% in var. Lollo Bionda at MAD 50% compared to the MAD 25% at harvest. But the reduction in yield in cv. Vera was around ~8.9% at MAD 50% compared to the MAD 25% at harvest. Also, at MAD 75%, the reduction in yield was 34% and 58% in cv. Vera and Lollo Bionda, respectively. Based on the interactive response between the cultivar and the deficit irrigation regimes in this study, it clearly shows that Vera is a well‐suited lettuce cultivar for deficit irrigation.

**Figure 2:**
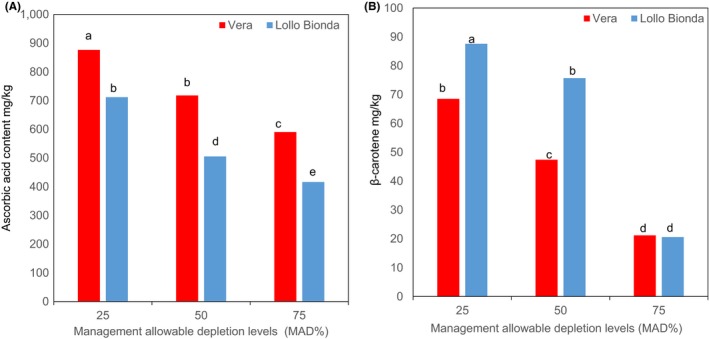
Effect of management allowable depletion levels (MAD%) on (A) ascorbic acid and (B) β‐carotene in green leafy lettuce varieties Vera and Lollo Bionda on dry weight basis. Management allowable depletion levels (MAD%): 25% (control), 50%, and 75%. Means in each bar for a specific parameter with the same letter are not significantly different at *p* < .05, Fisher's least significant difference (*n* = 12)

Interaction effect between the cultivar and irrigation on the ascorbic acid content was noted in this study (Figure 2A). The ascorbic acid content in lettuce leaves decreased with increasing water stress condition and declined significantly at MAD 75% (Figure 2A). Lettuce leaves from the control (MAD 25%) showed higher concentration of ascorbic acid. Ascorbic acid content was reduced to 18% and 30% in cultivars Vera and Lollo Bionda at water stress at MAD 50% compared to the control (25% MAD) (Figure 2A). At MAD 75%, the decline in the ascorbic acid content was around 32.6% in cv. Verra and 41.4% in cv. Lollo Bionda compared to their controls (Figure 2A). Similarly, lettuce plants subjected to multiple water stress showed significantly lower ascorbic acid content (Min, Carey, & Rajashekar, 2010). The concentration of β‐carotene declined in both cultivars when irrigated at MAD 75% (Figure 2B). At MAD 50%, β‐carotene accumulation in lettuce cv. Vera was reduced by 28.57%, while in cv. Bionda, the β‐carotene content was reduced by 11.36%, demonstrating that it is less affected by deficit irrigation (Figure 2B). Water stress has previously been shown to increase the β‐carotene concentration in different plant spp. (Min et al., 2010). However, in this study an interactive response between the deficit irrigation and the cultivar on β‐carotene was evident (Figure 2B).

**Figure 3:**
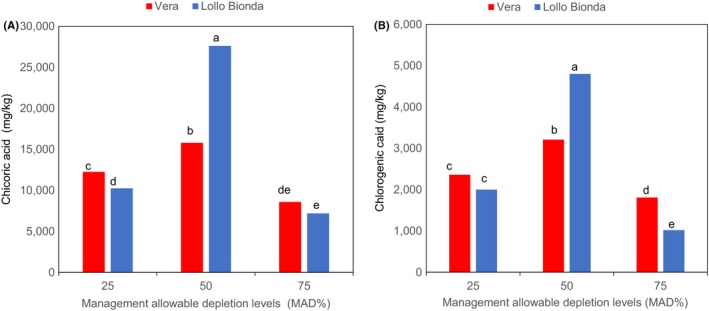
Effect of management allowable depletion levels (MAD%) on (A) chichoric and (B) chlorogenic acid content in green leafy lettuce varieties Vera and Lollo Bionda on dry weight basis. Management allowable depletion levels (MAD%): 25% (control), 50%, and 75%. Means in each bar for a specific parameter with the same letter are not significantly different at *p* < .05, Fisher's least significant difference (*n *= 12)

**Figure 4:**
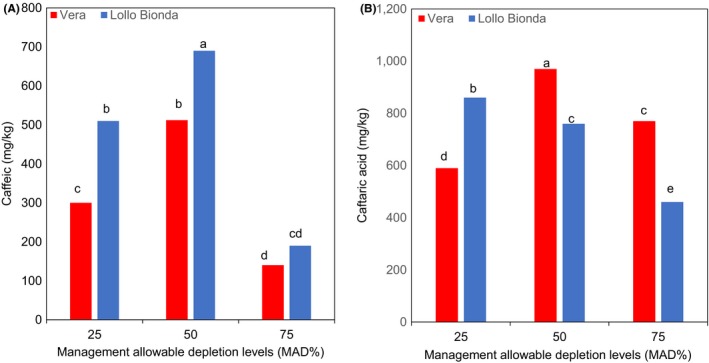
Effect of management allowable depletion levels (MAD%) on the concentrations of (A) caffeic and (B) caftaric acids content in green leafy lettuce varieties Vera and Lollo Bionda on dry weight basis. Management allowable depletion levels (MAD%): 25% (control), 50%, and 75%. Means in each bar for a specific parameter with the same letter are not significantly different at *p* < .05, Fisher's least significant difference (*n* = 12)

**Figure 5:**
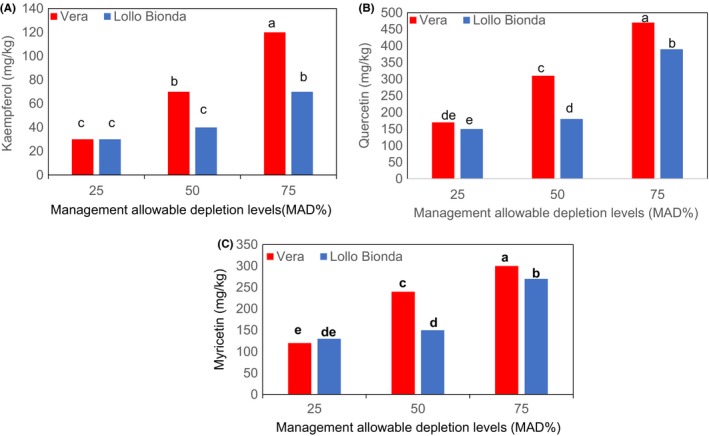
Effect of management allowable depletion levels (MAD%) on (A) kaempferol, (B) quercetin, and (C) myricetin content in green leafy lettuce varieties Vera and Lollo Bionda on dry weight basis. Management allowable depletion levels (MAD%): 25% (control), 50%, and 75%. Means in each bar for a specific parameter with the same letter are not significantly different at *p* < .05, Fisher's least significant difference (*n* = 12)

**Figure 6:**
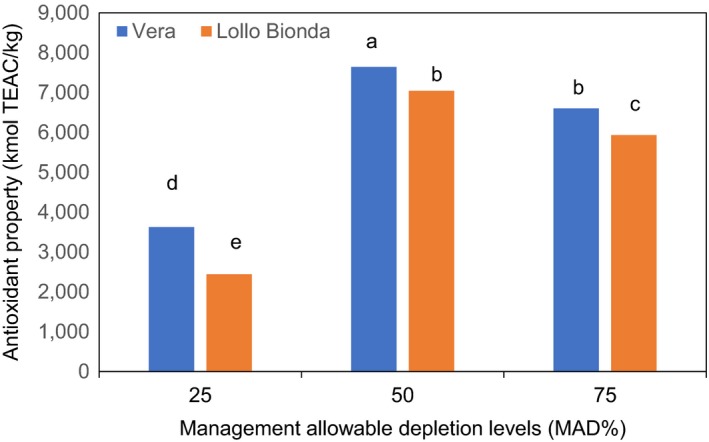
Effect of management allowable depletion levels (MAD%) on antioxidant property of green leafy lettuce varieties Vera and Lollo Bionda on dry weight basis. Management allowable depletion levels (MAD%): 25% (control), 50%, and 75%. Means in each bar for a specific parameter with the same letter are not significantly different at *p* < .05, Fisher's least significant difference (*n* = 12)

Similarly, an interactive effect was observed between the deficit irrigation and cultivars with respect to chicoric, chlorogenic, caftaric, and caffeic acids as shown in Figures 3A,B and 4A,B. The MAD 50% was shown to increase chicoric acid in cultivars Vera and Lollo Bionda at harvest (Figure 3A). However, cv. Lollo Bionda showed significantly higher chicoric acid (27,610 mg/kg dw) content than cv. Vera (15,800 mg/kg dw) at MAD 50%. Earlier findings showed that lettuce plants subjected to multiple water stress treatments showed higher chicoric acid content than the control plants (Min et al., 2010). It appears that chicoric acid concentration declined at MAD 75% in both cultivars and similar trend was observed with the caftaric, caffeic, and chlorogenic acid concentrations (Figures 3A,B and 4A,B). The MAD 50% improved the concentrations of chicoric, chlorogenic, and caffeic acids in both lettuce cultivars (Figures 3A,B and 4A,B). Also, the concentrations of caftaric in cultivars Vera Lollo and Bionda were highest at MAD 50% and 25% deficit irrigation regimes, respectively (Figure 4B). The trends on influence of deficit irrigation at 50% and 25% on concentration of phenolic acid components differed in both cultivars (Figures 3A,B and 4A,B). Previous studies have shown that irrigation regimes influence the composition of phenolic compounds (Luna et al., 2012). The same authors have shown that the concentration of chlorogenic acid reduced at deficit irrigation (R4 [301–400] mm with reduced available water) in iceberg lettuce. But the concentration of chicoric acid in iceberg lettuce increased at deficit irrigation (R4 [301–400] mm). It is also evident from our findings and the reports of Luna et al. (2012) that the influence of different deficit irrigation regimes on the components of phenolic acids differed remarkably. Chicoric and chlorogenic acid are major hydroxycinnamates in lettuce (Min et al., 2010). Chichoric acid is well known for many health benefits (Ivanov, 2014), and the antiobesity and antidiabetic effects of lettuce are linked to the chlorogenic acid (Cheng et al., 2014). Water stress induces the gene expression and the enzyme activity of phenylalanine ammonia lyase (PAL), the primary enzyme of phenylpropanoid pathway that is responsible for the biosynthesis of phenolic compounds (Min et al., 2010). The MAD at 75% resulted in a significant increase in concentrations of flavonols such as kaempferol, quercetin, and myricetin in both lettuce cultivars (Figure 5A–C). Water stress (abiotic stress) was demonstrated to induce the biosynthesis of flavonols synthesized via phenylpropanoid pathway by inducing transcription of genes encoding PAL, cinnamate‐4‐hydroxylase (C4H), 4‐coumarate: coenzyme A ligase (4CL), chalcone synthase (CHS), chalcone isomerase (CHI), and flavanone‐3‐hydroxylase (F3H) (Deluc et al., 2009). In recent years, flavonoid compounds are shown to have many health benefits. Ascorbic, chicoric, caftaric, caffeic, and chlorogenic acids, kaempferol, quercetin, myricetin, and β‐carotene are antioxidants. The interaction effect between the deficit irrigation levels and cultivars on antioxidants are shown in Figure 6. The antioxidant capacity (FRAP) increased with water stress and was significantly higher at MAD at 50% in both lettuce varieties (Figure 6), and this is likely due to the increased hydroxycinnamates as shown in Figures 3 and 4. On the contrary, findings of Min et al. (2010) showed the concomitant increase in total phenolic concentration and antioxidant capacity in water stressed lettuce plants.

**Figure 7:**
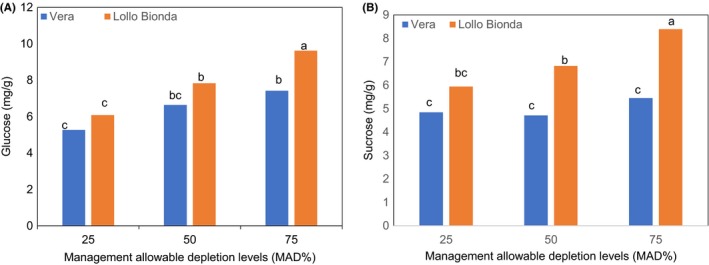
Effect of management allowable depletion levels (MAD%) on (A) glucose and (B) fructose content in green leafy lettuce varieties Vera and Lollo Bionda on dry weight basis. Management allowable depletion levels (MAD%): 25% (control), 50%, and 75%. Means in each bar for a specific parameter with the same letter are not significantly different at *p* < .05, Fisher's least significant difference (*n *= 12)

The interaction effect between the deficit irrigation levels and cultivars with respect to glucose and sucrose are shown in Figure 7A,B. Highest concentration of glucose and sucrose were noted in lettuce cv. Lollo Bionda at MAD 75% (Figure 7A,B. However, in cv. Vera, highest glucose concentration was obtained at MAD 50% and 75%. (Figure 7A). However, no significant variation was observed with respect to the different MAD levels and fructose concentration in cv. Vera (Figure 7B). Therefore, it is evident that the increase in glucose and fructose in cv. Lollo Bionda (leaves) shows that the plant cells were under water stress (Mills & Behboudian, 1996). Sucrose, glucose, and fructose are known as soluble sugars in plants. Gupta and Kaur (2005) explained that sucrose and glucose participate as substrates for cellular respiration or as osmolytes to maintain cell homeostasis. An increase in fructose is more related to the biosynthesis of secondary metabolites (phenolic compounds) (Hilal et al., 2004) than providing osmoprotection (Rosa et al., 2009). In this study, the following events, for example, the lower stomatal conductance (data not included) causing lower photosynthesis (Yakushiji et al., 1996), could have influenced the hydrolysis of sucrose due to the increased activity of invertase enzymes for the osmotic adjustments of turgor pressure maintenance in the plant. Furthermore, the signaling effect of endogenous abscisic acid could be responsible for the observed increase in glucose and fructose in the lettuce leaves (sink) (Kanayama, Moriguchi, Deguchi, Kanahama, & Yamaki, 2007). The increasing concentrations of phenolic compounds can also negatively affect the sensory properties (de Oliveira, de Carvalho, & Melo, 2014). Sugar accumulation in the leaves can also improve the taste and could overcome the negative impact of phenolic compounds on the taste of lettuce leaves.

Higher concentration of hydroxycinnamic acid derivatives could disadvantage the lettuce quality during fresh cut processing after postharvest storage by acting as substrates for the polyphenol oxidase enzyme activity and could result in browning or pinking. (Luna et al., 2012; Monaghan, Vickers, Grove, & Beacham, 2017). Phenolic acids namely chlorogenic acid, isochlorogenic acid, and chicoric acid (dicaffeoyl tartaric acid) were reported as substrates for the browning enzymes polyphenol oxidase (PPO) and peroxidase (POD) responsible for the browning in lettuce (Tomás‐Barberán et al., 1997). Previous findings of Luna et al. (2012) showed that the browning increased at lower deficit irrigation regimes due to the increase of caffeic acid derivatives that acted as substrates for the PAL enzyme activity. It can be speculated based on the effect of deficit irrigation regimes on caffeic acid concentrations in both cultivars that cv. Lollo Bionda could be susceptible for browning at MAD 50% than the cv. Vera. Furthermore, browning could be reduced remarkably at MAD 75% in both cultivars. However, in order to confirm further research need to be conducted with respect to the deficit irrigation regimes and PAL, PPO, and POD activities in order to investigate the impact of different phenolic acids on browning in these two lettuce cultivars.

## 4 CONCLUSIONS

Deficit irrigation has potential to improve the dietary phytochemicals in leafy lettuce cultivars and the phytochemicals have numerous health benefits. Higher deficit irrigation levels (MAD 75%) significantly reduced the substrates for browning reaction but negatively affected the marketable yield. Therefore, growers must balance the benefits in terms of quality (i.e., reducing browning) and the drawbacks due to the reduced salable weight to make appropriate decisions in implementation of deficit irrigation of 50% or 75% for marketing quality lettuce. Cultivar Vera is a well‐suited variety for management allowable depletion levels (50%) for producing desirable fresh mass and improved crop quality.

## CONFLICT OF INTEREST

None declared.
